# Bifidogenic Effect of 2′-Fucosyllactose (2′-FL) on the Gut Microbiome of Healthy Formula-Fed Infants: A Randomized Clinical Trial

**DOI:** 10.3390/nu17060973

**Published:** 2025-03-11

**Authors:** Tamara Lazarini, Karina Merini Tonon, Humberto Bezerra de Araujo Filho, Mauro Batista de Morais

**Affiliations:** 1 Nutrition Postgraduate Program, Universidade Federal de São Paulo, São Paulo 04023-062, Brazil; maurobmorais@gmail.com; 2 Department of Environmental & Public Health Sciences, University of Cincinnati College of Medicine, Cincinnati, OH 45267, USA; kari.tonon@gmail.com; 3 Division of Pediatric Gastroenterology, Universidade Federal de São Paulo, São Paulo 04023-062, Brazil; biogene@gmail.com

**Keywords:** gut microbiome, human milk oligosaccharides, breast milk, 2-fucosyllactose, galacto-oligosaccharides, fructo-oligosaccharides, infant formula

## Abstract

Breast milk is rich in bioactive components, especially human milk oligosaccharides (HMOs), which are crucial for establishing gut microbiota. The 2′-FL (2-Fucosyllactose), one of the most abundant oligosaccharides in breast milk, functions as a selective prebiotic. **Objective:** To examine the effect of adding 2′-FL (2-Fucosyllactose) to an infant formula containing prebiotic galacto-oligosaccharides (GOSs) and fructo-oligosaccharides (FOSs) on the gut microbiome of healthy formula-fed infants. **Methods**: This study enrolled infants from three groups: an HMO experimental group (*n* = 29), a GOS/FOS control group (*n* = 30), and an exclusively breastfed (breast milk [BM]) reference group (*n* = 28). Fecal samples from the three groups in the first and fourth months of life were analyzed. The V3 and V4 regions of the 16S rRNA gene were amplified and sequenced on the Illumina MiSeq. ANOVA, Kruskal–Wallis, richness indices (Chao1, Shannon), UniFrac distances, and the Adonis tests were used to perform statistical analyses on the relative abundance of phyla and genera, as well as the alpha and beta-diversity of the gut microbiota. **Results**: After intervention, Actinobacteriota emerged as the predominant phylum in both the HMO (60.4%) and BM (46.6%) groups. *Bifidobacterium* and *Escherichia-Shigella* were identified as the two most abundant bacterial genera in both groups. Nevertheless, the statistical analysis showed that the relative abundance of *Bifidobacterium* in the HMO formula-fed group after intervention was similar to that in the BM group (*p* > 0.05). Infants in the HMO and GOS/FOS groups showed higher relative abundance of *[Ruminococcus]_gnavus_group* bacteria compared to those in the BM group. Groups fed with infant formula demonstrated higher alpha-diversity of gut microbiota compared to breastfed infants (*p* < 0.05), at the time of admission as well as after the intervention. Beta-diversity was significantly different among the three groups, according to type of feeding. Infants fed a 2′-FL-supplemented infant formula exhibited growth comparable to that of breastfed infants throughout the intervention period, demonstrating that the formula was both safe and well tolerated. **Conclusions**: Adding 2′-FL to an infant formula containing 4 g/L of GOS + FOS resulted in a stronger bifidogenic effect compared to the formula without 2′-FL.

## 1. Introduction

Breast milk is unquestionably the optimal nutrition for infants. Exclusive breastfeeding is recommended for the first 6 months, followed by continued breastfeeding alongside complementary foods until 24 months or longer [1,2,3]. However, in cases where exclusive or partial breastfeeding is not possible, infant formulas are considered the best alternatives for infants, in line with guidelines from pediatric and nutrition societies [3,4,5].

Oligosaccharides constitute the third most abundant solid component in human milk, following lactose and lipids, with their concentration in mature milk ranging from 5 to 15 g/L [5,6]. The set of human milk oligosaccharides (HMOs) comprises more than 200 molecules present at varying concentrations, with 2′-fucosyllactose (2′-FL) being one of the most abundant, reaching concentrations on the order of grams per liter [5,6,7,8], playing a key role in gut microbiota development, promoting mutualists and eliminating potential pathogens [5,6,8,9,10]. The gut microbiota develops rapidly after birth, thoroughly directed by breast milk [7,10].

The gut microbiome of breastfed infants differs from formula-fed infants, particularly when the formula lacks oligosaccharides. The gut microbiota of breastfed infants is dominated by *Bifidobacterium* and *Lactobacillus*, while formula-fed infants have a more diverse microbiota with about 40–60% *Bifidobacterium* and *Lactobacillus*, and the remaining portion comprising *Enterobacteriaceae* and *Bacteroides* [11,12]. HMO has been associated with the presence of *Bifidobacterium* in the infant gut microbiome [13,14].

The specific effect of each HMO molecule in the gut microbiota installation has been investigated lately but the available information is still limited. The simple structure of the 2′-FL molecule enables its synthesis through chemical, enzymatic, and biotechnological processes, making it one of the first HMOs to be synthetically produced on a large scale [15]. As one of the most abundant HMOs in breast milk and offering beneficial properties, 2′-FL has been studied in both experimental and clinical research [16].

Experimental studies have shown that α1-2 fucosylated HMO, particularly 2′-FL, potentially inhibit infections and colonization by pathogens in the gut microbiota, including *Escherichia coli*, rotavirus, norovirus, and *Campylobacter jejuni*. *Campylobacter jejuni* is a primary cause of bacterial diarrhea, which is a major factor in child mortality [9,17]. Moreover, 2′-FL has been identified as the primary nutrient for symbiotic bacteria isolated from the gut microbiome of breastfed infants, including *Bifidobacterium longum subsp. infantis*, *Bifidobacterium bifidum*, and *Bacteroides fragilis* [18,19]. It is important to note that HMOs, such as 2′-FL, demonstrate a highly selective prebiotic effect. They are preferentially consumed by specific *Bifidobacterium* and *Bacteroides* species in the infant’s intestine [20,21].

Clinical studies on adding 2′-FL and Lacto-N-neotetraose (LNnT) to infant formula showed good tolerance and acceptance by infants [22,23,24]. The combination of 2′-FL and LNnT helped modulate gut microbiota composition, reduce infections, and decrease medication use [25].

The incorporation of HMOs into infant formulas is becoming increasingly prevalent, supported by a growing body of clinical evidence demonstrating its benefits. However, due to vast structural and functional diversity of HMOs, further research is needed to determine the optimal combination of oligosaccharides for use in infant formula. Specifically, combinations of HMOs with other prebiotics, such as GOSs and FOSs, are under-investigated. Additionally, adding 2′-FL to a GOS-containing infant formula enhanced its immune-modulating properties (reducing inflammatory cytokines), although its effect on gut microbiota has not been studied [26]. Notably, GOS + FOS combinations have been standard in infant formulas for decades. Adding GOS and FOS to infant formula has a bifidogenic effect that is well demonstrated [5,27]. On the other hand, research on adding 2′-FL to these prebiotic-enhanced formulations is limited.

In this context, the present study aimed to evaluate the effect of adding 2′-FL (2-Fucosyllactose) to an infant formula containing a combination of prebiotics (4 g per liter of GOS + FOS) on the composition of the gut microbiota in healthy infants. The gut microbiota of exclusively breastfed infants was used as reference.

## 2. Materials and Methods

### 2.1. Study Design

A randomized, double-blind, controlled trial with two formula-fed groups and a prospective, observational companion study of a subsample that investigated the gut microbiota profile of the participating infants. The gut microbiota composition of formula-fed infants was analyzed at two time points: at enrollment (less than 1 month old) and after 3 months of intervention (approximately 4 months old). Infants formula-fed were randomly divided into two groups randomized: 1. The HMO group, received a standard formula containing 5 g/liter of oligosaccharides of 2′-FL + GOS [Galacto-oligosaccharide] and FOS [Fructo-oligosaccharide] combination in a 9:1 ratio, as the experimental group. 2. The GOS/FOS group received a standard formula containing 4 g/liter of a GOS [Galacto-oligosaccharide] and FOS [Fructo-oligosaccharide] combination in a 9:1 ratio, as the control group. The two infant formulas were nutritionally identical, with bovine milk-based whey predominant formula with an energy density of 670 kcal/L containing 75 g/L lactose, 34 g/L fat, and 14 g/L intact protein (60:40 whey: casein ratio), differing only by 2′-FL molecule addition. Randomization was categorized as simple and conducted using a dynamic allocation algorithm. Parents, caregivers, investigators, and study support staff were blinded to the identity of the study formulas, using two meaningless codes (1 per formula group) provided by the manufacturer (Nestle Product Technology Center, Konolfingen, Switzerland). The nutritional composition of experimental and control infant formulas are available on Supplementary Methods. Findings from the experimental and control groups were also compared against a reference group of exclusively breastfed infants (BM). The relative abundance of fecal microbiota and bacterial diversity were evaluated at enrollment and after 90 days of nutritional intervention, a period that allows the assessment of the dynamic effects of colonization and modulation of the infant gut microbiota. This study was approved by the ethics committee of the Universidade Federal de São Paulo and registered at clinicaltrials.gov as RBR-7g2d86j.

### 2.2. Study Population

From August 2020 to March 2023, infants were enrolled in pediatric clinics in three Brazilian cities, Recife, São Paulo, and Porto Alegre, each one representing a different geographic region. The following inclusion criteria were considered for enrollment in the study: 1. Healthy full-term infants (37–42 weeks) weighing 2500–4500 g at birth. 2. Being formula-fed without the possibility of resuming natural breastfeeding, according to the assessment of the pediatricians responsible for monitoring the study. All inclusion criteria were similar to the BM reference group, except that exclusive breastfeeding from birth until the second stool sample (collected 90 days after enrollment) was required.

The sample size (*n* = 90) was derived from a subsample of a primary study. However, a specific sample size calculation for molecular biology analyses of the intestinal microbiota was not performed due to the lack of validated parameters in the literature.

Infants attended study visits at baseline (≤30 days of age) and subsequently at three monthly medical visits. At baseline, demographic and anthropometric characteristics, medical history, and birth data were recorded. At each visit, anthropometric measurements, including weight, length, and head circumference, were assessed.

Fecal samples were collected at baseline (before the intervention started) and between 3 and 4 months of age, depending on when the infant was admitted. Parent-reported and physician-confirmed adverse events.

To monitor infant growth and development, weight gain between baseline and 4 months of age in formula-fed infants was measured as the primary outcome, following the guidelines of the American Academy of Pediatrics Task Force on Clinical Testing of Infant Formulas. Secondary outcomes included anthropometric z-scores, stool characteristics, and gastrointestinal (GI) tolerance as well as parent-reported and physician-confirmed adverse effects [28].

Infant weight was measured without clothing or a diaper using a calibrated electronic scale, with precision to the nearest 10 g. Recumbent length was recorded using a calibrated length board, accurate to the nearest 0.1 cm, and head circumference was measured with a standard non-elastic measuring tape to the nearest 0.1 cm. Corresponding z-scores for weight-for-age, length-for-age, weight-for-length, and head circumference-for-age were calculated using the World Health Organization (WHO) Child Growth Standards [29].

**Figure 1:**
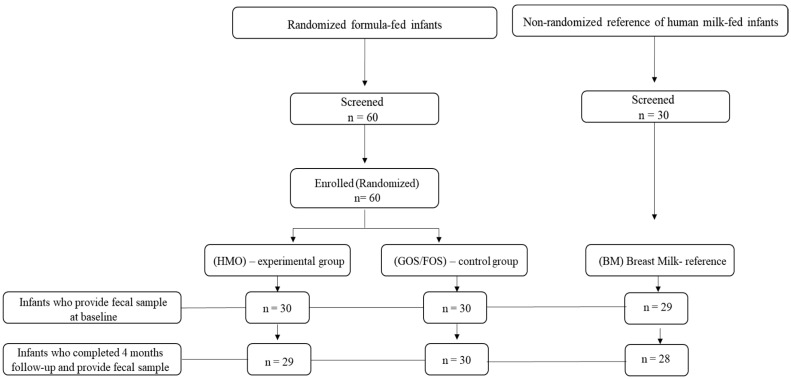
Subject disposition for the randomized controlled trial with formula-fed infants and the companion study with human milk-fed infants serving as the reference group. (HMO) experimental group, (GOS/FOS) control group, and (BM) breast milk-fed infants.

Exclusion criteria included the following: mixed feeding methods, potential resumption of breastfeeding, introduction of complementary foods before the second stool sample collection, congenital diseases or defects, other serious pre-existing conditions, current antibiotic use, and parents under 18 years of age. Figure 1 shows the distribution of infants into three groups for this study.

### 2.3. Gut Microbiota Analysis

#### 2.3.1. Collection and Storage

During routine check-ups, pediatricians collected non-invasive stool samples by extracting approximately 1 g of fecal matter from diapers when patients had their first and second fecal collection. Using a disposable spatula, the samples were transferred to Zymo Research DNA/RNA Shield™ R1101 fecal collection tube. The tube contains a stabilizing solution that preserves DNA for subsequent 16S rRNA analysis. DNA/RNA Shield Fecal Collection tubes are designed for the collection and preservation of nucleic acids from fecal samples. These stool collection tubes create a microbial snapshot while deactivating viruses, ensuring samples are safe and ready for shipping [30]. Details are available on Supplementary Methods.

The collected samples were stored at −80 °C in a centralized facility managed by the Diagnostics of America S.A (DASA) Group from Sao Paulo, Brazil. DASA then forwarded these samples to Biomehub Research and Development Ltd. from Santa Catarina, Brazil for molecular biology assays.

#### 2.3.2. Microbiota Analysis

DNA extraction was conducted using the ZymoBIOMICS DNA Miniprep kit, following the manufacturer’s protocol as previously described. The V3/V4 region of the 16S rRNA gene was amplified in a two PCR system (primers V3/V4: 341F CCTACGGGRSGCAGCAG and 806R GGACTACHVGGGTWTCTAAT).

Unnormalized amplicon libraries were pooled and quantified via Qubit fluorometry (Invitrogen, Waltham, MA, USA) and qPCR using the Kapa Library Quantification Kit (Kapa Biosystems, Woburn, MA, USA).

Sequencing was conducted on the MiSeq equipment (Illumina Inc., San Diego, CA, USA) using the V2x300 kit, single-end, 300 bp. Sequences were imported and analyzed using QIIME2 software (version 2023.5), then subjected to quality control via the DADA2 package to eliminate sequencing errors and chimeric sequences. Subsequently, sequences were clustered into operational taxonomic units (OTUs) to create frequency tables for each sample. The taxonomic identification of each OTU was conducted using the SILVA-ARB_version 138 database as a reference [30,31].

**Figure 2:**
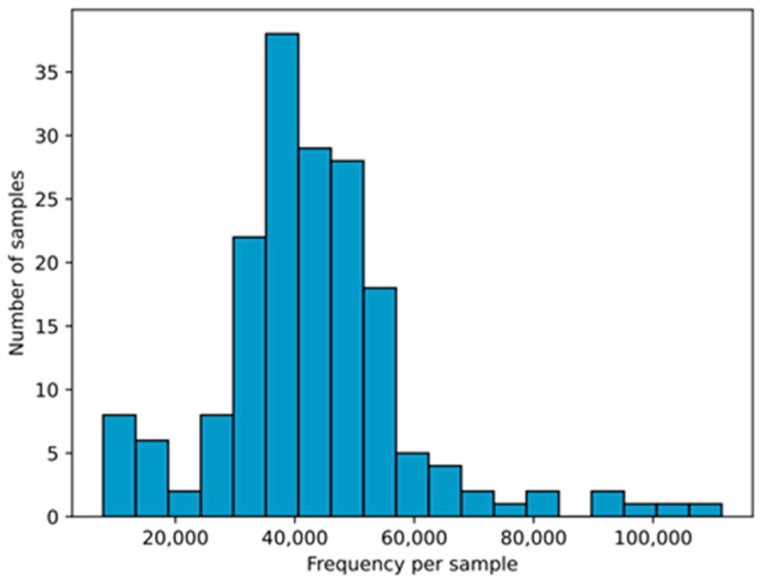
Histogram of the number of DNA sequences in the samples (*n* = 178) from the three groups included in the study, collected at admission and after the intervention period.

This study analyzed 178 fecal samples, initially yielding 11,679,205 DNA sequences. After filtering and removing chimeras, 7,651,734 sequences remained, identifying 912 OTUs across 12 bacterial phyla and 169 genera. The median found was 41,261.5 (34,769.25–50,303.5) per sample according to Figure 2.

#### 2.3.3. Statistical Analysis and Bioinformatics

One-way analysis of variance (ANOVA) was used to assess differences in the demographic and anthropometric characteristics of infants. Additionally, infant growth and development were evaluated using anthropometric indices expressed as percentiles and z-scores, including weight-for-length (W/L), weight-for-age (W/A), length-for-age (L/A), head circumference-for-age (HC/A), and body mass index-for-age (BMI/A). These indices were compared with the World Health Organization (WHO) growth curves [29].

The data were analyzed using XLStat version 2018.6 (São Paulo, Brazil) and SigmaPlot version 14 (São Paulo, Brazil). Qualitative data were reported as absolute frequencies and percentages. Quantitative data were presented as means and standard deviations or medians and interquartile ranges, depending on each variable’s distribution. We also applied the Kruskal-Wallis test to analyze variations in the relative abundance of major phyla and genera, as well as the alpha-diversity of infant fecal microbiota. This was complemented by Dunn’s test for multiple comparisons. Alpha-diversity was evaluated using Chao1 and Shannon indices to measure species richness. Beta-diversity was quantified using weighted and unweighted UniFrac distances, analyzed via the Adonis multivariate method.

This study was approved by the Research Ethics Committee of the São Luiz Hospital Maternity under the number 4.155.728 and of the Federal University of São Paulo—UNIFESP under the number 5.118.107.

## 3. Results

### 3.1. Demographic and Anthropometric Characteristics and Fecal Microbiota Profile of Infants at Study Enrollment

Table 1 presents the characterization of the studied population, including variables such as age, sex, gestational age, mode of delivery (vaginal or cesarean), and anthropometric measurements (weight, length, and head circumference).

At admission, the three groups were comparable in terms of sex, age, birth weight, and gestational age.

The proportion of vaginal deliveries was higher among exclusively breastfed (BM) infants (55.2%) compared to those fed HMO formula (37.9%) and GOS/FOS formula (36.7%). However, this difference was not statistically significant (*p* = 0.280).

**Figure 3:**
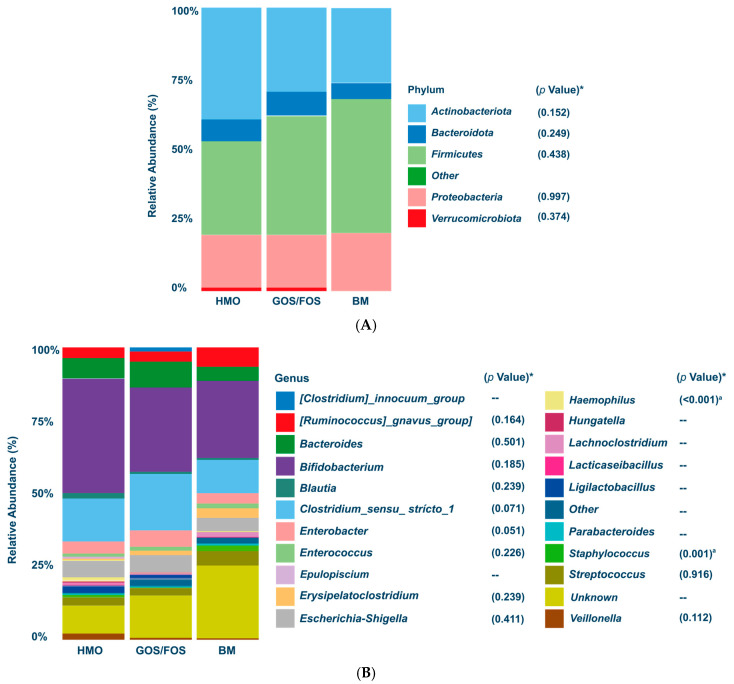

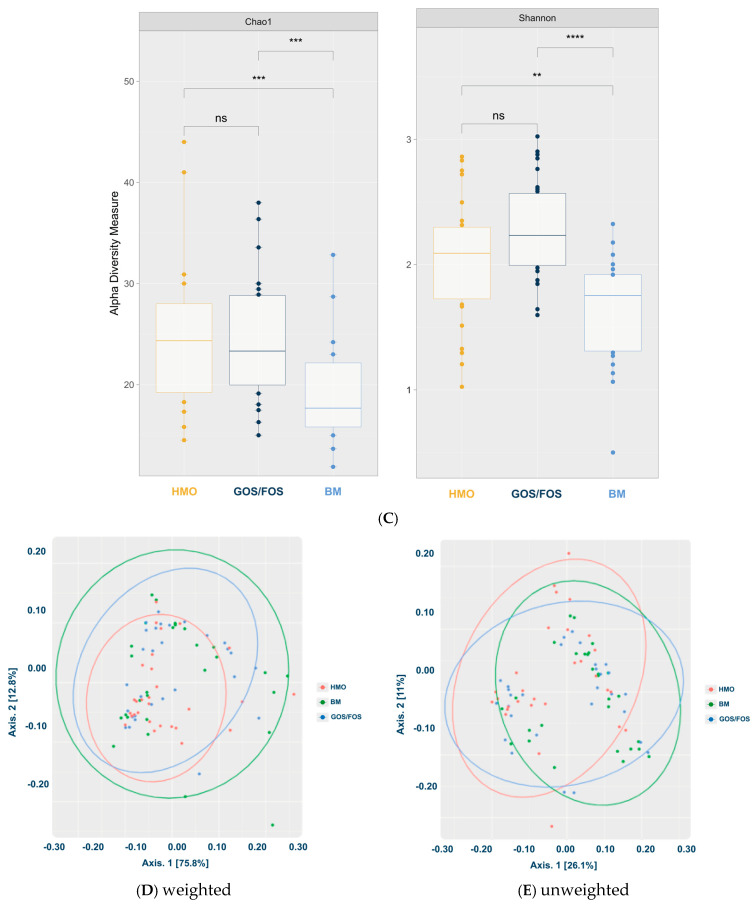
Upon enrollment: Panel (**A**) Relative abundance of the most prevalent phyla. (*) = *p* values. Panel (**B**) Relative abundance of the most prevalent genera. (*) = *p* values. Kruskal–Wallis test followed by Dunn’s test for multiple comparisons: a. HMO x GOS/FOS *p* > 0.05; **HMO x BM *p* < 0.05; GOS/FOS x BM *p* < 0.05**. Panel (**C**) Graphical representation (“boxplot”) of alpha-diversity using Chao1 and Shannon indices, with the Kruskal–Wallis test followed by Dunn’s test for multiple comparisons. ns = not significant; ** *p* < 0.01 for Shannon index HMO x BM; *** *p*< 0.01 for Chao1 index HMO x BM and GOS/FOS x BM; **** *p*< 0.01 for Shannon index GOS/FOS x BM. Panel (**D**) is the final weighted UniFrac distance by feeding type (*p* = 0.48); sex (*p* = 0.57); delivery mode (*p* < 0.001 *). Panel (**E**) Final unweighted UniFrac distance by feeding type (*p* = 0.004 *), sex (*p* = 0.01 *), and delivery mode (*p* < 0.02 *). (*) = statistical significance. HMO, experimental group; GOS/FOS, control group; BM, breast milk-fed infants.

Figure 3A shows no statistically significant difference in the relative abundance of phyla at study enrollment. Figure 3B shows that the BM group has a higher relative abundance of *Haemophilus* and *Staphylococcus.* Figure 3C shows lower alpha diversity Chao1 and Shannon indices in the BM group than in the other two groups. Additionally, we have included Supplementary Tables presenting the median values of bacterial phyla and genera and alpha-diversity at admission and at the end of the study in the Supplementary Results: Tables S1–S6.

Beta-diversity, analyzed using weighted and unweighted Principal Coordinate Analysis (PcoA), considered feeding type, sex, and mode of delivery. Figure 3D shows that the weighted analysis reached statistical significance for the delivery method (*p* < 0.01), whereas in the unweighted analysis (Figure 3E), all included parameters showed statistically significant differences.

### 3.2. Dietary Intervention and Anthropometric Changes

The randomized groups (HMO or GOS/FOS) were exclusively formula-fed, while the reference group (BM) was exclusively breastfed for the entire study period. Table 2 presents the intervention duration and changes in anthropometric measurements. Across all three groups, the parameters examined showed no significant differences.

According to the primary outcomes, all infants were classified as eutrophic after the intervention period based on nutritional status assessment [29], with growth and development within the normal range for age, defined by percentile values between 3 and 97 and a Z-score between −2 SD and +2 SD. However, the HMO group exhibited a significantly higher weight-for-age percentile (W/A) compared to the BM group. (More details in Supplementary Results: Table S7).

### 3.3. Fecal Microbiota Profile After Intervention

#### 3.3.1. Relative Abundance of Bacterial Phyla and Genera

**Figure 4:**
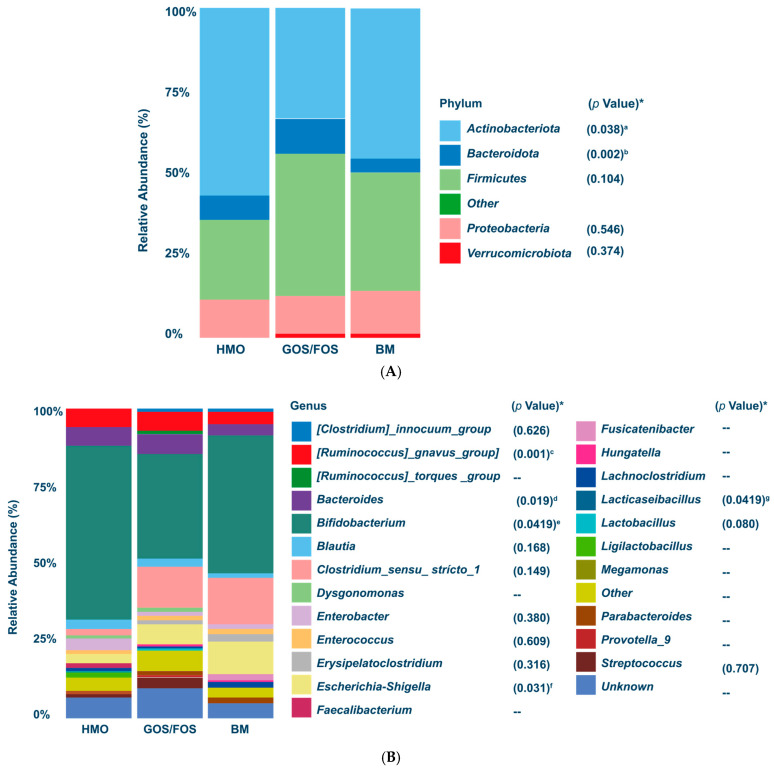

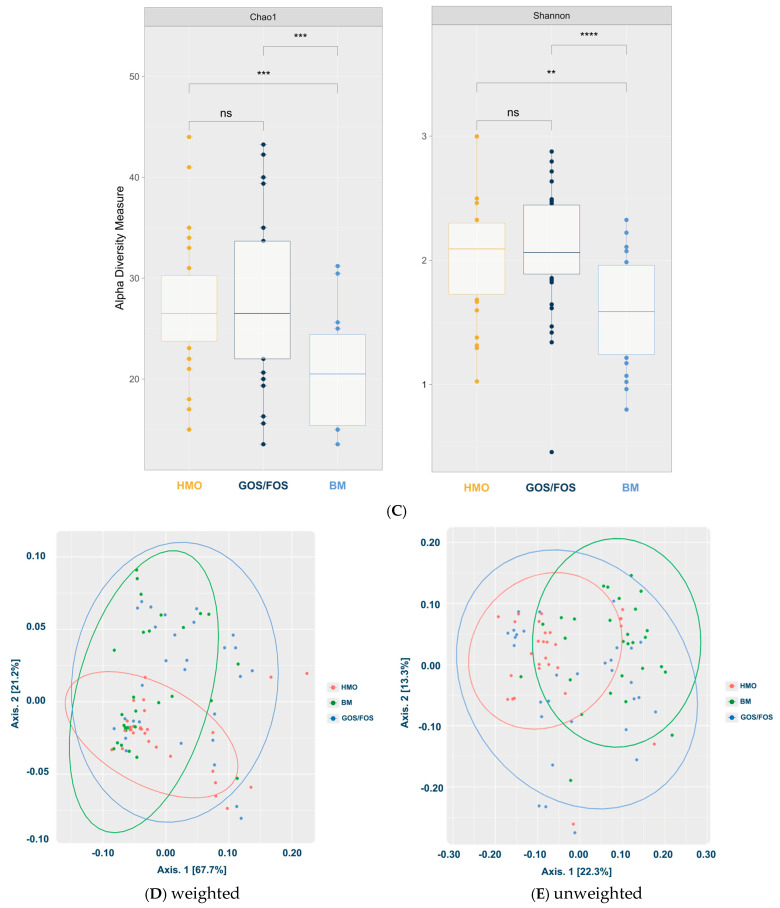
At the end of the study: Panel (**A**) Relative abundance of the most prevalent phyla. (*) = *p* values. The Kruskal–Wallis test followed by Dunn’s test for multiple comparisons: **a. HMO x GOS/FOS *p* < 0.05**; HMO x BM *p* > 0.05; BM x GOS/FOS *p* > 0.05; **b.** HMO x GOS/FOS *p* > 0.05; **HMO x BM *p* < 0.05**; BM x GOS/FOS *p* > 0.05. Panel (**B**) Relative abundance of the most prevalent genera. (*) = *p* values. The Kruskal–Wallis test followed by Dunn’s test for multiple comparisons: **c.** HMO x GOS/FOS *p* > 0.05; **HMO x BM *p* < 0.05; BM x GOS/FOS *p* < 0.05; d.** HMO x GOS/FOS *p* > 0.05; **HMO x BM *p* < 0.05; BM x GOS/FOS *p* < 0.05; e. HMO x GOS/FOS *p* < 0.05**; HMO x BM *p* > 0.05; BM x GOS/FOS *p* > 0.05; **f**. HMO x GOS/FOS *p* > 0.05; **HMO x BM *p* < 0.05;** BM x GOS/FOS *p* > 0.05; g. HMO x GOS/FOS *p* > 0.05; HMO x BM *p* > 0.05; **BM x GOS/FOS *p* < 0.05**. Panel (**C**) Graphical representation (“box-plot”) of alpha-diversity using Chao1 and Shannon indices, with the Kruskal–Wallis test followed by Dunn’s test for multiple comparisons. ns = not significant; ** *p* < 0.01 for Shannon index HMO x BM; *** *p* < 0.01 for Chao1 index HMO x BM and GOS/FOS x BM; **** *p* < 0.01 for Shannon index GOS/FOS x BM. Panel (**D**) Final weighted UniFrac distance by type of feeding (*p* < 0.004 *); sex (*p* = 0.60); delivery mode (*p* = 0.45). Panel (**E**) Final unweighted UniFrac distance by feeding type (*p* = 0.001 *); sex (*p* = 0.45); delivery mode: (*p* = 0.81). (*) = statistical significance. HMO, experimental group; GOS/FOS, control group; BM, breast milk-fed infants.

The results showed that Actinobacteriota had the highest relative abundance in the HMO (60.4%) and BM (46.6%) groups, while Firmicutes had the highest relative abundance in the GOS/FOS group (Figure 4A). The *Kruskal–Wallis* test showed that the HMO group had a significantly higher relative abundance of Actinobacteriota phylum (*p* < 0.05) compared to the GOS/FOS group. In contrast, no statistically significant difference was found between the HMO and GOS/FOS groups and the reference BM group. The HMO group showed a higher relative abundance of the Bacteroidota phylum compared to the BM group.

Figure 4B demonstrates that *Bifidobacterium* emerged as the dominant bacterial genus in all three groups by the end of the study. The HMO group showed significantly higher (*p* < 0.05) *Bifidobacterium* abundance compared to the GOS/FOS group. However, Dunn’s multiple comparison test revealed no significant difference in the relative abundance of *Bifidobacterium* between the HMO and GOS/FOS groups when compared to the BM group. Compared to the BM group, the HMO and GOS/FOS groups had higher relative abundance of *[Ruminococcus]_gnavus_group* and *Bacteroides* genera. Meanwhile, the genus *Escherichia-Shigella* was higher in the BM group than in the HMO group. Ultimately, the BM group had a higher relative abundance of the *Lacticaseibacillus* genus than the GOS/FOS group.

#### 3.3.2. Alpha-Diversity and Beta-Diversity in the Final Collection

The BM group had lower fecal microbiota alpha-diversity at the study’s end than the HMO and GOS/FOS groups, with *p* < 0.001 for both Chao1 and Shannon indices (Figure 4C). Beta-diversity analysis using weighted (Figure 4D) and unweighted (Figure 4E) UniFrac distances showed no differences based on delivery mode or infant sex. However, both matrices revealed statistically significant differences when examining feeding type by group (*p* = 0.004 and *p* < 0.001, respectively).

#### 3.3.3. Beta-Diversity: Pairwise Comparison Between Groups After Intervention

To better elucidate the results pertaining to fecal microbiota beta-diversity at the end of intervention, a Principal Coordinate Analysis (PcoA) was conducted. This analysis employed both weighted and unweighted UniFrac for pairwise comparisons among the studied groups.

**Figure 5:**
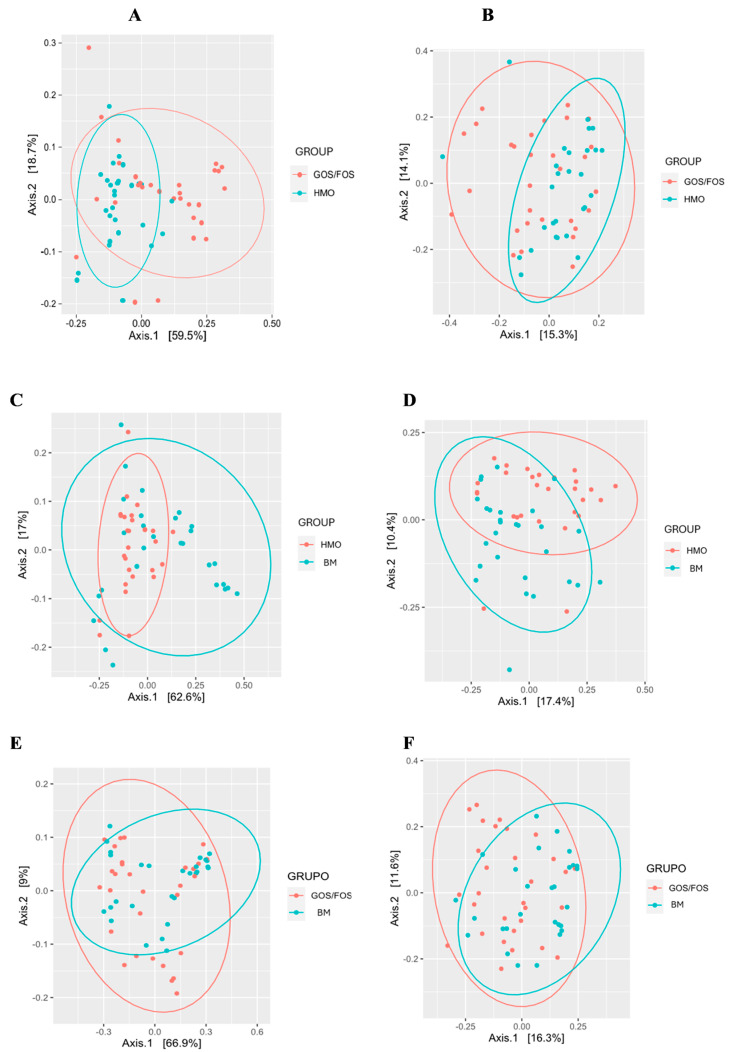
Beta-diversity: pairwise comparison between groups after intervention. (*) = statistical significance. Panel (**A**) UniFrac distance weighted at the end according to HMO x GOS/FOS groups *p* = 0.002 *. Panel (**B**) Final unweighted UniFrac distance for HMO vs. GOS/FOS groups *p* = 0.013 *. Panel (**C**) Final weighted UniFrac distance for HMO vs. BM groups *p* = 0.009 *. Panel (**D**) Final unweighted UniFrac distance for HMO vs. BM groups *p* = 0.001 *. Panel (**E**) Final weighted UniFrac distance according to the GOS/FOS x BM groups *p* = 0.185 *. Panel (**F**) Final unweighted UniFrac distance for GOS/FOS vs. BM groups *p* = 0.002 *. HMO, experimental group; GOS/FOS, control group; BM, Breast milk-fed infants.

Figure 5 shows statistically significant differences in the comparisons between the HMO and GOS/FOS groups (Panels A and B) and between the HMO and BM groups (Panels C and D), based on both weighted and unweighted UniFrac distances. The weighted UniFrac distance did not differ between the GOS/FOS and BM groups (Panel E). In contrast, the unweighted UniFrac distance (Panel F) revealed a statistically significant difference between these groups.

#### 3.3.4. Gastrointestinal Symptoms and Bowel Habits of Infants

As secondary outcomes, Table 3 presents the gastrointestinal and behavioral symptoms of infants recorded over three consecutive days prior to the final sample collection. For the presence or absence of regurgitation, flatulence, excessive crying, irritability, and sleep, Pearson’s chi-square test was applied, showing no significant differences between the groups. However, according to multiple comparisons using the Kruskal–Wallis test, followed by Dunn’s test, infants in the BM group had a higher number of regurgitation and flatulence episodes within 24 h. Infants in the HMO group, compared to the other groups, exhibited a shorter duration of irritability and more hours of sleep per night.

At the end of the study, intestinal habits, including bowel movement frequency, evacuation effort, and stool consistency, were similar among the three groups (Table 4).

## 4. Discussion

Our research demonstrated that infants fed formula with the HMO 2′-fucosyllactose had higher relative abundance of *Bifidobacterium* (59.5%) than those given formula containing only GOS + FOS (24.4%). The findings also revealed that the relative abundance of *Bifidobacterium* was statistically similar (*p* > 0.05) to that of the exclusively breastfed reference group (46.6%). A higher abundance of *Bifidobacterium* is considered beneficial for formula-fed infants since this genus exerts a series of immunomodulatory functions in the neonate [32]. *Bifidobacterium* species and strains demonstrate a superior ability to assimilate and metabolize HMOs favoring their growth to the detriment of pathogens [14,32,33,34,35]. Our finding aligns with a study of ninety-four exclusively breastfed infants, where *Bifidobacterium* was the most abundant genus in fecal samples of infants up to 6 months of age, comprising an average relative abundance of 70% of the microbiota [36]. A study examining the relationship between HMO concentrations in breast milk and infant gut microbiota composition demonstrated that higher levels of 2′-FL and LNFP1 were associated with an increased relative abundance of *Bifidobacterium* [36]. The interactions among various species and strains within bifidobacterial communities contribute to shaping the diversity and prevalence of *Bifidobacterium* in infants during early life [37]. It is important to note that bifidobacteria species vary in their ability to use HMOs. *Bifidobacterium breve*, prevalent in infant gut microbiome, typically cannot metabolize 2′-FL. Additional metagenomic analyses showed that extracellular “fucosidases” encoded by co-occurring microorganisms, such as *Ruminococcus gnavus*, initiate the metabolism of 2′-FL, and the released lactose promotes the exponential growth of *Bifidobacterium breve*. In conclusion, the use of HMOs may vary based on other components of an individual’s microbiome [38]. In this context, our study showed a higher relative abundance of *Ruminococcus gnavus* in formula-fed groups compared to exclusively breastfed infants. Future clinical studies should be designed to investigate the targeted effects of 2′-FL on various bifidobacterial species in the gut microbiota. In this context, an in vitro study using the SHIME^®^ digestive simulator demonstrated that the relative abundance of *Bifidobacteriaceae* was greater following 2′-FL fermentation by infant microbiota compared to lactose. This effect was observed in both 3-month-old infants and children aged 2–3 years [39]. Another study, employing the SHIME^®^ digestive simulator to examine four distinct oligosaccharide profiles (Lactose, 2′-FL, 2′-FL + LNnT, and a combination of 6 HMO molecules), demonstrated that the strong and immediate bifidogenic effect of HMOs was primarily due to the stimulation of *Bifidobacterium adolescentis* and, to a lesser extent, *Bifidobacterium dentium*, *Bifidobacterium bifidum*, and *Bifidobacterium longum*. Specifically, the group receiving infant formula containing a combination of six oligosaccharides had concurrent increases in both *Bifidobacterium adolescentis* and *Faecalibacterium prausnitzii* species. *Faecalibacterium prausnitzii* is a major butyrate producer, which is associated with the development of a healthy gut microbiota [40]. The study concluded that more complex HMO combinations could promote diverse bifidobacteria colonies in infants’ guts and help prevent pro-inflammatory imbalances in the gut mucosa [40]. It should be noted that the effects of HMOs are not limited to the gut microbiota. A clinical study reported that infants (*n* = 200) fed for 6 weeks on infant formula supplemented with 2′-FL + GOS demonstrated lower levels of plasma inflammatory cytokines than infants who received formula with GOSs but without the addition of HMOs [26].

Research has long highlighted differences in gut microbiota establishment between exclusively breastfed infants and those fed formula. However, caution is warranted when reviewing studies on infant formulas, as their formulations and clinical outcomes can differ based on the type of prebiotics added such as FOSs, GOSs, PDX, and various HMOs [27]. In this context, a study in China and Mongolia examined ninety-one infants, comparing the gut microbiome of healthy infants who were either exclusively breastfed or given formula without HMOs. At 40 days old, alpha-diversity was lower in breastfed groups than in formula-fed groups, but it increased significantly by 6 months of age. *Bifidobacterium* emerged as the prevalent genus, with *Enterobacteriaceae* ranking second in all groups. After 40 days, the breastfed group showed significantly higher levels of *Bifidobacterium* and *Bacteroides*, but lower levels of Streptococcus and *Enterococcus* compared to infants fed formula without HMOs [41]. Studies show that formula-fed infants tend to have a relatively higher abundance of *Escherichia coli*, *Bacteroides*, and *Clostridium difficile* than breastfed infants [42,43]. However, the relative abundance of Actinobacteria phylum and *Bifidobacterium* spp. is similar in infants, regardless of whether they are breastfed or formula-fed [44].

This study found that at 4 months, the Actinobacteriota phylum was dominant in the HMO and BM groups, while bacteria from the Firmicutes phylum prevailed in the GOS/FOS group. The relative abundance of Bacteroidota was higher in the HMO group than in the BM group. After a 3-month nutritional intervention, *Lactobacillus*, *Lacticaseibacillus*, and [*Clostridium]_innocuum_group* genera emerged in all three groups, despite being absent at study enrollment. This demonstrates the dynamic physiological process of gut microbiota establishment during this life stage. Infants fed formula showed higher levels of *Bacteroides* and the *[Ruminococcus]_gnavus_group* compared to breastfed infants. The genus *Bifidobacterium* was more prevalent in the HMO group than in the GOS/FOS group but showed no statistically significant difference from the BM group. Finally, the genera *Lacticaseibacillus* and *Escherichia-Shigella* showed higher relative abundance in the BM group than in the GOS/FOS and HMO groups, respectively. Some of our findings aligned with those from the KOALA cohort study in the Netherlands. Their PCR analysis of fecal samples from 1032 infants revealed that, at one month of age, exclusively formula-fed infants had higher colonization rates of *Escherichia coli*, *Clostridium difficile*, *Bacteroides*, and *Lactobacillus* than breastfed infants [43].

Our results showed that both formula-fed groups showed higher alpha-diversity compared to the breastfed group, both at enrollment and at the end of the intervention. Infants on formula alone have more diverse gut bacteria than those who are breastfed, including fewer beneficial *Bifidobacterium* [45] and more Firmicutes and *Bacteroides* [14]. A review summarized these findings, showing higher microbial alpha-diversity in formula-fed infants than in those who were breastfed. In contrast, it revealed a greater abundance of *Bifidobacterium* in exclusively breastfed infants [46]. A United Kingdom cohort study [36] examined infant gut microbiota development in ninety-one exclusively breastfed infants. The study also revealed an age-related biphasic pattern: gut microbiota alpha-diversity decreased from 2 weeks to 3 months, then increased up to 6 months of age. Our study revealed that breastfed infants demonstrated an evolutionary increase in alpha-diversity at 1 and 4 months of age. However, this alpha-diversity was statistically lower in both samplings compared to formula-fed infants. Conversely, a European study found a contrasting microbiological finding. Infants fed formula supplemented with 2′-FL + LNnT showed fecal microbiota diversity similar to breastfed infants, unlike those fed formula without HMOs [23,25]. The low bacterial diversity in breastfed infants may be partially explained by the selective prebiotic effect of HMOs, since only a limited variety of species can utilize HMOs for growth [7,10,47].

Our study revealed that the mode of delivery was associated with variations in beta diversity at enrollment, which disappeared after the intervention. However, the final analysis showed that beta-diversity differences, based on weighted and unweighted UniFrac distances, were exclusively associated with diet type.

Beyond their influence on gut microbiota establishment and immune function, there is growing interest in HMOs’ potential effects on infant growth. This study evaluated anthropometric measurements such as weight, length, and head circumference. Infants in all three groups demonstrated age-appropriate development. The addition of up to 1 g/L of 2′-FL in an infant formula with 4 g/L of GOS + FOS (ratio 9:1), did not result in any differences in infant weight or growth compared to other groups, showing good tolerance and safety.

The formula-fed groups (HMO and GOS/FOS) exhibited similar gastrointestinal symptoms, except for the HMO group, which had a shorter duration of irritability and more hours of sleep per night compared to the GOS/FOS group.

Bowel habits, including frequency, evacuation effort, and stool consistency, were comparable among the three groups at both the beginning and end of the study.

A key strength of this study is the recruitment of infants from three major cities across northeast, southeast, and south regions of Brazil, a country with extensive territory. Thus, the study population better represents Brazilian demographics. To the best of our knowledge, this is the first clinical study to evaluate the combination of these oligosaccharides on the infant gut microbiota in Latin America. Another key strength was the low dropout rate (3.3%; 3/90) for infants completing the follow-up and gut microbiota assessments, enabling more robust and reliable statistical analysis. Throughout the study, several limitations were observed, which can be highlighted, such as the need for a more detailed clinical anamnesis, including a thorough investigation of the infants’ feeding history prior to study admission; information on maternal education, income, and family history; habitual diet and maternal nutritional status; and the presence of gestational complications or diseases. These variables would have enabled further analyses and associations. Another potential limitation of this study and similar research is the inherent difficulty in interpreting the effects of HMOs on infant microbiota, which are overly complex and challenging to investigate. Thus, we employed a widely accepted sequencing methodology and comprehensively detailed bioinformatics techniques to ensure our results are comparable with existing and future studies. In the present study, we applied 16S rRNA sequencing to identify bacterial phyla and genera. However, species identification was not included, as it would require greater financial investment for a new shotgun metagenomic sequencing. An identified opportunity would be the collection of breast milk samples from the mothers of infants in the exclusively breastfed group, which would allow for expanded analyses correlating the profile and quantity of maternal HMOs with the infant’s microbiota.

Studies on human milk oligosaccharides have emerged as a promising field of scientific research, not only for infant studies but also for other stages of life, such as childhood and adulthood.

Research on HMOs has emerged as a promising field, not only for studies involving infants but also for other stages of life, including childhood and adulthood. There is still much to be explored regarding the potential effects of oligosaccharides on human health, particularly in shaping the gut microbiota during the first 2 to 3 years of life, when it stabilizes and becomes similar to adult gut microbiota. Consequently, investing in metagenomic research and its potential correlations with inter- and intra-individual factors is becoming increasingly crucial.

## 5. Conclusions

Infants fed a 2′-FL-supplemented infant formula exhibited growth comparable to that of breastfed infants throughout the intervention period, demonstrating that the formula was both safe and well tolerated.

The addition of 2′-FL to an infant formula containing prebiotics (4 g/L of GOS + FOS) enhanced its bifidogenic effect compared to a formula without 2′-FL. This bifidogenic effect positively contributes to the modulation of gut microbiota during childhood. Statistical analysis revealed that, following the intervention, the relative abundance of *Bifidobacterium* in the HMO formula-fed groups was similar to that observed in the exclusively breastfed group (*p* > 0.05).

Although numerous studies have explored the role of oligosaccharides in infancy, few have specifically examined their influence on the intestinal microbiota, particularly in Brazilian infants. Therefore, further clinical research in this area is of paramount importance. The findings of this study may serve as a foundation for future investigations.

## Figures and Tables

**Table 1:** Demographic and anthropometric characteristics of the three study groups at birth and at study enrollment.

Characteristics	GROUP			*p* ^1,2^
	HMO (*n* = 29)	GOS/FOS (*n* = 30)	BM (*n* = 29)	
**Infant age (days)**				
Enrollment	16.9 ± 4.62	16.7 ± 6.21	17.1 ± 5.25	0.953
**Sex**				0.81
Female	15 (51.7%)	13 (43.3%)	14 (48.3%)	
Male	14 (48.3%)	17 (56.7%)	15 (51.7%)	
**Gestational age (weeks)**	38.7 ± 1.10	38.6 ± 0.95	39.0 ± 1.07	0.316
**Delivery mode**				0.28
Vaginal	11 (37.9%)	11 (36.7%)	16 (55.2%)	
C-Section	18 (62.1%)	19 (63.3%)	13 (44.8%)	
**Weight (grams)**				
Birth	3155.10 ± 318.24	3178.80 ± 342.59	3140.2 ± 353.40	0.907
Enrollment	3253.40 ± 376.40	3309.00 ± 367.04	3191.2 ± 387.34	0.489
**Length (cm)**				
Birth	49.1 ± 1.78	49.1 ± 1.59	48.7 ± 1.26	0.624
Enrollment	50.1 ± 1.87	50.3 ± 1.58	49.9 ± 1.35	0.595
**Head circumference (cm)**				
Birth	34.1 ± 0,88	34.5 ± 0.98	34.3 ± 1.09	0.292
Enrollment	35.0 ± 0.91	35.5 ± 1.07	35.1 ± 1.01	0.132
**Body Mass Index (BMI)**				
Birth	13.0 ± 0.71	13.1 ± 0.82	13.2 ± 1.04	0.854
Enrollment	12.9 ± 0.77	13.0 ± 0.95	12.8 ± 1.01	0.596

^1^. Values expressed as mean and ±SD (standard deviation). One-way analysis of variance, ANOVA. ^2^. The chi-square statistical test was employed to analyze sex and delivery mode. HMO, experimental group; GOS/FOS, control group; BM, breast milk-fed infants.

**Table 2:** Anthropometric measurements and incremental values according to the three study groups at the end of the study.

Characteristics	GROUP			*p* ^1^
	HMO (*n* = 29)	GOS/FOS (*n* = 30)	BM (*n* = 29)	
**Infant age (days)**				
Final	111.8 ± 6.20	111.0 ± 6.44	112.1 ± 6.61	0.786
**Interval duration (days)**	94.9 ± 5.35	94.3 ± 6.64	94.9 ± 7.09	0.899
(Minimum and maximum)	85–105	85–110	85–109	
**Weight (grams)**				
Final	6190.30 ± 311.56	6068.80 ± 458.68	6000.70 ± 514.27	0.251
Incremental ^2^	2936.90 ± 363.09	2759.80 ± 352.60	2809.60 ± 570.33	0.287
**Length (cm)**				
Final	61.10 ± 1.50	61.00 ± 1.50	60.80 ± 1.46	0.798
Incremental ^2^	10.99 ± 1.51	10.68 ± 1.70	10.94 ± 1.80	0.745
**Head Circumference (cm)**				
Final	40.3 ± 0.92	40.5 ± 0.97	40.4 ± 1.11	0.824
Incremental ^2^	5.37 ± 0.84	5.02 ± 1.09	5.32 ± 1.09	0.364
**Body Mass Index (BMI)**				
Final	16.6 ± 0.50	16.2 ± 0.95	16.2 ± 1.03	0.194
Incremental ^2^	3.66 ± 1.03	3.22 ± 1.23	3.42 ± 1.20	0.351

^1^. Values expressed as mean and ± SD (standard deviation). One-way analysis of variance, ANOVA. ^2^. Incremental values of anthropometric measurements (final measurement—admission measurement). HMO, experimental group; GOS/FOS, control group; BM, breast milk-fed infants.

**Table 3:** Gastrointestinal and behavioral symptoms of infants recorded during three consecutive days prior to the final collection, according to the groups investigated.

Symptoms/ Behavior ^1^ (per Day)	GROUP			*p* ^3,4^
	HMO (*n* = 29)	GOS/FOS (*n* = 30)	BM (*n* = 29)	
**Regurgitation**				0.082
No	17 (58.6%)	16 (53.3%)	09 (31%)	
Yes	12 (41.4%)	14 (46.7%)	20 (69%)	
Number of times/day ^2^	0.0 (0.0–2.0)	0.0 (0,0–3.0)	1.0 (0,0–3.0)	0.003 ^a^
Volume (mL) ^2^	5.0 (5.0–5.0)	5.0 (5.0–5.0)	15.0 (5.0–30.0)	<0.001 ^b^
**Flatulence**				0.218
No	18 (62.1%)	18 (60%)	12 (41.4%)	
Yes	11 (37.9%)	12 (40%)	17 (58.6%)	
Number of times/day ^2^	0.0 (0.0–2.0)	0.0 (0.0–3.0)	1.0 (0.0–3.0)	0.016 ^c^
**Excessive Crying**				0.108
No	19 (65.5%)	16 (53.3%)	11 (37.9%)	
Yes	10 (34.5%)	14 (46.7%)	18 (62.1%)	
Time (minutes) ^2^	0.0 (0.0–10.0)	0.0 (0.0–15.0)	0.0 (0.0–15.0)	0.047 ^d^
**Irritation/Agitated**				0.125
No	23 (79.3%)	18 (60%)	16 (55.2%)	
Yes	06 (20.7%)	12 (40%)	13 (44.8%)	
Time (minutes) ^2^	0.0 (0.0–10.0)	0.0 (0.0–20.0)	0.0 (0.0–15.0)	<0.001 ^e^
**Sleep**				
Hours/night ^2^	15.0 (11.0–17.0)	12.0 (4.0–15.0)	12.0 (10.0–15.0)	<0.001 ^f^

^1^. Diary of gastrointestinal symptoms and behavior. Record of 3 consecutive days prior to the consultation with the pediatrician in the final collection. ^2^. Values expressed as median and 25th and 75th percentiles. ^3^. Pearson’s chi-square test for continuous variables. ^4^. Kruskal–Wallis test, supplemented by Dunn’s test for multiple comparisons: a. **HMO x BM *p* < 0.05;** HMO x GOS/FOS *p* > 0.05; BM x GOS/FOS *p* > 0.05; b. **HMO x BM *p* < 0.05;** HMO x GOS/FOS *p* > 0.05; **BM x GOS/FOS *p* < 0.05**; c. **HMO x BM *p* < 0.05;** HMO x GOS/FOS *p* > 0.05; BM x GOS/FOS *p* > 0.05; d. HMO x BM *p* > 0.05; HMO x GOS/FOS *p* > 0.05; BM x GOS/FOS *p* > 0.05; e. **HMO x BM *p* < 0.05; HMO x GOS/FOS *p* < 0.05;** BM x GOS/FOS *p* > 0.05; f. **HMO x BM *p* < 0.05; HMO x GOS/FOS *p* < 0.05;** BM **x** GOS/FOS *p* > 0.05.

**Table 4:** The bowel habits of infants recorded over the three consecutive days prior to the final collection, according to the investigation groups.

Manifestations ^1^	GROUP			*p* ^2,4^
	HMO (*n* = 29)	GOS/FOS (*n* = 30)	BM (*n* = 30)	
**Defecation**				0.861
No	03 (10.3%)	02 (6.7%)	03 (10%)	
Yes	26 (89.7%)	28 (93.3%)	27 (90%)	
**Frequency ^3^**				
Number of times/day	2.0 (2.0–3.0)	3.0 (1.0–4.2)	3.0 (1.0–5.0)	0.455
**Straining during defecation**				0.81
No	22 (84.6%)	25 (90.1%)	24 (90.5%)	
Yes	04 (15.4%)	03 (9.9%)	03 (9.5%)	
**Fecal Consistency ^5^**				0.262
Unformed	23 (88.5%)	22 (79.1%)	22 (82.1%)	
Formed	03 (11.5%)	06 (20.9%)	17 (17.9%)	

^1^. Diary of fecal manifestations. Record of three consecutive days prior to the final pediatric consultation. ^2^. Pearson’s chi-square test for continuous variables. ^3^. Values expressed as median and 25th and 75th percentiles. ^4^. Kruskal–Wallis test. ^5^. Stool consistency categorized as unformed (watery and/or soft) and formed (formed and/or hard).

## Data Availability

Data described in the manuscript, code book, and analytic code will be made available upon request pending application and approval. The data are not publicly available due to privacy and ethical reasons.

## Supplementary Materials

The following supporting information can be downloaded at https://www.mdpi.com/article/10.3390/nu17060973/s1. Supplementary Methods and Supplementary Results. References [30,48,49,50,51] are cited in supplementary files.

## Abbreviations

The following abbreviations are used in this manuscript:

2′-FL	2-Fucosyllactose
FOS	Fructo-oligosaccharides
GOS	Galacto-oligosaccharides
GOS/FOS group	Control group
HMO	Human milk oligosaccharides
HMO group	Experimental group
BM group	Breast milk group (reference)
