# Supplementary material for: Bifidogenic Effect of 2′-Fucosyllactose (2′-FL) on the Gut Microbiome of Healthy Formula-Fed Infants: A Randomized Clinical Trial
Source: Nutrients. 2025 Mar 11;17(6):973. doi: 10.3390/nu17060973 (PMC11944528; doi:10.3390/nu17060973)
Supplement: Supplementary file 1 [file nutrients-17-00973-s001.zip › Supplementary Methods_Bifidogenic effect of 2'-fucosyllactose.pdf]

Bifidogenic effect of 2'-fucosyllactose (2'-FL) on the gut microbiome of healthy formula-fed infants

Tamara Lazarini et al.

Online supplementary material

Supplementary Methods

Table of Contents

Nutritional composition of infant formulas considered in the study 2
Guidelines for collecting fecal samples 4
References 5

Table S1. Nutritional composition of infant formulas considered in the study[1]

Component	Control Formula[2] (Nan Comfor 1, Nestlé®)	Experimental Formula[2] (Nestlé®)
Calories	67 kcal	67 kcal
Carbohydrates	7,2 g	7,2 g
Protein	1,3 g	1,3 g
Total fat	3,7 g	3,7 g
Saturated fat	1,4 g	1,4 g
Trans fat	0 g	0 g
Linoleic acid	0,5 g	0,5 g
α-linolenic acid	63 mg	63 mg
Docosahexaenoic acid (DHA)	7,0 mg	7,0 mg
Arachidonic acid (ARA)	7,0 mg	7,0 mg
**Prebiotics**	**0,4 g**	**0,5 g**
**Fructo-oligosaccharides (FOS)**	**0,04 g**	**0,04 g**
**Galacto-oligosaccharide (GOS)**	**0,4 g**	**0,4 g**
**2´-Fucosyl-Lactose (2´-FL)**	**-**	**0,1 g**
Sodium	17 mg	17 mg
Calcium	43 mg	43 mg
Iron	0,73 mg	0,73 mg
Potassium	64 mg	64 mg
Chloride	49 mg	49 mg
Phosphorus	23 mg	23 mg
Magnesium	8,4 mg	8,4 mg
Zinc	0,76 mg	0,76 mg
Manganese	15 µg	15 µg
Iodine	16 µg	16 µg
Copper	53 µg	53 µg
Selenium	1,8 µg	1,8 µg
Vitamin A	61 µg RE	61 µg RE

Vitamin D	1,3 µg	1,3 µg
Vitamin E	0,93 mg α TE	0,93 mg α TE
Vitamin K	6,0 µg	6,0 µg
Vitamin C	11 mg	11 mg
Vitamin B1	0,08 mg	0,08 mg
Vitamin B2	0,12 mg	0,12 mg
Niacin	0,57 mg	0,57 mg
Vitamin B6	0,04 mg	0,04 mg
Vitamin B12	0,13 µg	0,13 µg
Folic acid	10 µg	10 µg
Pantothenic acid	0,48 mg	0,48 mg
Biotin	1,9 µg	1,9 µg
Choline	7,3 mg	7,3 mg
Inositol	7,3 mg	7,3 mg
Taurine	4,4 mg	4,4 mg
L-carnitine	1,3 mg	1,3 mg
Nucleotides	2,1 mg	2,1 mg

[1] *Values per 100 ml of ready-to-use reconstituted formula.*
[2] *Infant formulas developed exclusively for Brazilian infants.*

Guidelines for collecting fecal samples

Protocol: FECAL COLLECTION TUBES

You are receiving a DNA/RNA Shield fecal collection tube, which ensures sample stability during storage and transportation at room temperature, without the need for refrigeration or specialized equipment. Along with the tube, you are receiving a plastic envelope with your baby's identification label.

Materials received:

1. Fecal collection tube
2. Plastic envelope for storing the collected material

When and how to collect?

- ✓ 1 to 3 days before the next appointment with the pediatrician
- ✓ Prerequisite: exclusive feeding with breast milk or formula. Cannot have ingested other types of food.
- ✓ Infant's age: up to 4 months old.

Instructions:

1. Prepare and collect the stool sample using the stool sample collection kit provided directly from your baby’s diaper.

Note: The method of collecting the stool sample should prevent stool from falling into the toilet water to prevent contamination of the sample.

2. Unscrew the cap from the collection tube and collect a spoonful of stool (approximately 1 gram or 1 ml in volume).
3. Place the sample in the collection tube.
4. Tighten the cap and SHAKE to mix the contents well (invert 10 times) to create a suspension.

Note: Foaming during shaking is normal.

5. Discard unused fecal material and wash your hands thoroughly.
6. Place the tube in the plastic bag and seal it by removing the blue tape.
7. Label the plastic bag with the date and time of collection.
8. Store at room temperature (no need to refrigerate or freeze).
9. Take it to your pediatrician at your next appointment.

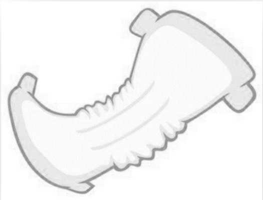

Collect feces directly from your baby's diaper

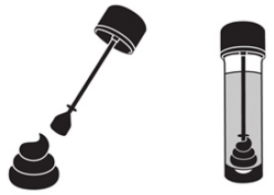

Take a spoonful and put it in the tube

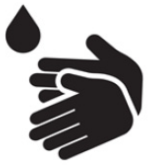

Wash your hands

**In most cases, feces were collected in the doctor's office.*

References

1. BRASIL. Resolução RDC no 43, de 19 de setembro de 2011. Aprova o regulamento técnico que estabelece os requisitos mínimos de identidade, composição, qualidade e segurança a que devem obedecer às fórmulas infantis para lactentes. DOU no 182, de 21 de setembro de 2011 vol. 2011 https://www.ibfan.org.br/site/wp-content/uploads/2014/06/Resolucao_RDC_n_43_de_19_de_setembro_de_2011.pdf (2011).
2. BRASIL. Resolução RDC no 44, de 19 de setembro de 2011. Aprova o regulamento técnico que estabelece os requisitos mínimos de identidade, composição, qualidade e segurança a que devem obedecer às fórmulas infantis de seguimento para lactentes e crianças de primeira infância. DOU no 182, de 21 de setembro de 2011 vol. 2011 https://bvsms.saude.gov.br/bvs/saudelegis/anvisa/2011/res0044_19_09_2011.html (2011).
3. Regulation (EC) No 258/97 of the European Parliament and of the council of 27 January 1997 concerning novel foods and novel food ingredients. Available online: http://data.europa.eu/eli/reg/1997/258/oj
4. Microbiomics by Zimo Catalog. Disponível em: 2019-2020_catalog_-_low_res.pdf zymoresearch.com. Available online: https://zymoresearch.eu/pages/literature
5. International Committee of Medical Journal Editors. Uniform requirements for manuscripts submitted to biomedical journals: writing and editing for biomedical publication [Internet]. Philadelphia (PA): ICMJE Secretariat office, American College of Physicians; [updated 2008 Oct; cited 2021 Mar 12]. Available from: http://www.icmje.org
