# Supplementary material for: Bifidogenic Effect of 2′-Fucosyllactose (2′-FL) on the Gut Microbiome of Healthy Formula-Fed Infants: A Randomized Clinical Trial
Source: Nutrients. 2025 Mar 11;17(6):973. doi: 10.3390/nu17060973 (PMC11944528; doi:10.3390/nu17060973)
Supplement: Supplementary file 1 [file nutrients-17-00973-s001.zip › Supplementary Results_Bifidogenic effect of 2'-fucosyllactose.pdf]

Bifidogenic effect of 2'-fucosyllactose (2'-FL) on the gut microbiome of healthy formula-fed infants

Tamara Lazarini et al.

Online supplementary material

Supplementary Results

Table of Contents

Table S1. Relative abundance of the most prevalent phyla identified in the fecal microbiota at baseline in the three study groups …………………02

Table S2. Relative abundance of the most prevalent phyla identified in the fecal microbiota after the intervention in the three study groups …….03

Table S3. Relative abundance of the most prevalent genera identified in the fecal microbiota at baseline in the three study groups ………………04

Table S4. Relative abundance of the most prevalent genera identified in the fecal microbiota after the intervention in the three study groups ……05

Table S5. Alpha-diversity indices in the fecal microbiota at baseline collection in the three studied groups …………………………………………06

Table S6. Alpha-diversity indices in the fecal microbiota at the final collection in the three studied groups …………………………………………06

Table S7. Percentile indices and Z-scores related to the anthropometric measurements of the investigated groups at the end of the study ……..07

Table S1. Relative abundance of the most prevalent phyla identified in the fecal microbiota at baseline in the three study groups

Phylum	GROUP			
	**HMO (n=29)**	**GOS/FOS (n=30)**	**BM (n=28)**	**p[2]**
Actinobacteriota	43.80 (9.39 – 62.10)	15.60 (0.17 – 62.7)	8.25 (0.03 – 51.9)	0.152
Bacteroidota	0.00 (0.00 – 11.4)	0.00 (0.00 – 8.78)	0.00 (0.00 – 0.01)	0.249
Firmicutes	22.80 (14.00 – 60.2)	35.10 (11.00 – 63.30)	49.40 (13.10 – 79.50)	0.438
Proteobacteria	13.00 (4.72 – 33.50)	9.32 (4.92 – 36.30)	12.70 (3.57 – 34.90)	0.997
Verrucomicrobiota	0.00 (0.00 – 0.00)	0.00 (0.00 – 0.00)	0.00 (0.00 – 0.00)	0.374

1. Values expressed as median and 25th and 75th percentiles. 2. Kruskal-Wallis test.

Table S2. Relative abundance of the most prevalent phyla identified in the fecal microbiota after the intervention in the three study groups

Phylum	GROUP			
	**HMO (n=29)**	**GOS/FOS (n=30)**	**BM (n=28)**	**p[2]**
Actinobacteriota	60.40 (46.70 – 68.30)	24.50 (2.91 – 55.90)	46.60 (5.21 – 82.70)	0.038[a]
Bacteroidota	5.57 (0.11 – 14.50)	1.36 (0.00 – 14.40)	0.00 (0.00 – 0.07)	0.002[b]
Firmicutes	18.00 (11.10 – 32.50)	42.40 (14.90 – 67.80)	20.50 (7.28 – 63.80)	0.104
Proteobacteria	5.63 (2.81 – 17.20)	7.64 (3.89 – 17.70)	8.83 (3.76 – 13.60)	0.546
Verrucomicrobiota	0.00 (0.00 – 0.00)	0.00 (0.00 – 0.11)	0.00 (0.00 – 0.00)	0.258

1. Values expressed as median and 25th and 75th percentiles.
2. Kruskal-Wallis test, followed by Dunn’s test for multiple comparisons:
   a. **HMO x GOS/FOS $p<0.05$**; HMO x BM $p>0.05$; BM x GOS/FOS $p>0.05$;
   b. HMO x GOS/FOS $p>0.05$; **HMO x BM $p<0.05$**; BM x GOS/FOS $p>0.05$.

Table S3. Relative abundance of the most prevalent genera identified in the fecal microbiota at baseline in the three study groups

GENUS	GROUP			
	HMO (n=29)	GOS/FOS (n=30)	BM (n=28)	p^2
[Ruminococcus]_gnavus	0.00 (0.00 – 7.20)	0.00 (0.00 – 0.50)	0.00 (0.00 – 0.00)	0.164
Bacteroides	0.00 (0.00 – 10.6)	0.00 (0.00 – 6.20)	0.00 (0.00 – 0.00)	0.501
Bifidobacterium	43.60 (14.20 – 61.50)	15.30 (0.00 – 59.30)	8.20 (0.00 – 49.50)	0.185
Blautia	0.00 (0.00 – 0.00)	0.00 (0.00 – 0.20)	0.00 (0.00 – 0.00)	0.239
Clostridium_sensu_stricto_1	3.30 (1.40 – 1.16)	8.00 (0.20 – 28.30)	0.20 (0.00 – 13.10)	0.071
Enterobacter	0.80 (0.00 – 6.50)	1.40 (0.40 – 5.80)	0.00 (0.00 – 3.30)	0.051
Enterococcus	0.50 (0.10 – 1.90)	0.30 (0.00' – 1.10)	0.00 (0.00 – 1.00)	0.226
Erysipelatoclostridium	0.00 (0.00 – 0.00)	0.00 (0.00 – 0.50)	0.00 (0.00 – 0.00)	0.239
Escherichia-Shigella	1.10 (0.00 – 4.00)	2.00 (0.00 – 6.00)	0.30 (0.00 – 5.40)	0.411
Haemophilus	0.00 (0.00 – 0.00)	0.00 (0.00 – 0.00)	0.10 (0.00 – 0.40)	<0.001[a]
Staphylococcus	0.00 (0.00 – 0.00)	0.00 (0.00 – 0.10)	0.10 (0.00 – 0.60)	0.001[b]
Streptococcus	1.20 (0.20 – 3.40)	0.80 (0.30 – 3.00)	0.50 (0.20 – 3.50)	0.916
Veillonella	0.30 (0.00 – 1.00)	0.40 (0.10 – 0.90)	0.10 (0.00 – 0.30)	0.112

1. Values expressed as median and 25th and 75th percentiles.
2. Kruskal-Wallis test, followed by Dunn's test for multiple comparisons:
   a. HMO x GOS/FOS p>0.05; **HMO x BM p<0.05; GOS/FOS x BM p<0.05;**
   b. HMO x GOS/FOS p>0.05; **HMO x BM p<0.05; GOS/FOS x BM p<0.05.**

Table S4. Relative abundance of the most prevalent genera identified in the fecal microbiota after the intervention in the three study groups

GENUS	GROUP			
	**HMO (n=29)**	**GOS/FOS (n=30)**	**BM (n=28)**	**p[2]**
[Clostridium]_innocuum_group	0.00 (0.00 – 0.10)	0.00 (0.00 – 0.20)	0.00 (0.00 – 0.00)	0.626
[Ruminococcus]_gnavus	2.30 (0.90 – 7.00)	1.80 (0.00 – 8.90)	0.00 (0.00 – 0.50)	0.001[a]
Bacteroides	2.00 (0.00 – 10.0)	0.00 (0.00 – 5.80)	0.00 (0.00 – 0.00)	0.019[b]
Bifidobacterium	59.50 (46.90 – 66.20)	24.40 (4.50 – 53.40)	46.60 (6.70 – 82.20)	0.0419[c]
Blautia	0.00 (0.00 – 1.60)	0.00 (0.00 – 1.20)	0.00 (0.00 – 0.00)	0.168
Clostridium_sensu_stricto_1	1.10 (0.20 – 4.20)	3.60 (0.60 – 17.00)	2.60 (0.40 – 19.40)	0.149
Enterobacter	0.10 (0.00 – 1.20)	0.70 (0.00 – 1.60)	0.00 (0.00 – 1.00)	0.380
Enterococcus	0.70 (0.10 – 1.80)	0.30 (0.10 – 1.40)	0.30 (0.10 – 1.00)	0.609
Erysipelatoclostridium	0.00 (0.00 – 0.10)	0.00 (0.00 – 1.20)	0.00 (0.00 – 0.10)	0.316
Escherichia-Shigella	2.50 (0.80 – 4.20)	4.00 (1.50 – 7.20)	5.00 (1.90 – 11.30)	0.031[d]
Lacticaseibacillus	0.00 (0.00 – 0.00)	0.00 (0.00 – 0.00)	0.00 (0.00 – 0.30)	0.0419[e]
Lactobacillus	0.00 (0.00 – 0.00)	0.00 (0.00 – 0.00)	0.00 (0.00 – 0.10)	0.080
Streptococcus	0.40 (0.10 – 1.30)	0.20 (0.00 – 1.40)	0.20 (0.00 – 1.10)	0.707

1. Values expressed as median and 25th and 75th percentiles.
2. Kruskal-Wallis test, followed by Dunn's test for multiple comparisons:
   a. HMO x GOS/FOS p>0.05; **HMO x BM p<0.05; BM x GOS/FOS p<0.05;**
   b. HMO x GOS/FOS p>0.05; **HMO x BM p<0.05; BM x GOS/FOS p<0.05;**
   c. **HMO x GOS/FOS p<0.05**; HMO x BM p>0.05; BM x GOS/FOS p>0.05;
   d. HMO x GOS/FOS p>0.05; **HMO x BM p<0.05;** BM x GOS/FOS p>0.05;
   e. HMO x GOS/FOS p>0.05; HMO x BM p>0.05; **BM x GOS/FOS p<0.05.**

Table S5. Alpha-diversity indices in the fecal microbiota at baseline collection in the three studied groups

	GROUP			
**Indice**	**HMO (n= 29)**	**GOS/FOS (n= 30)**	**BM (n= 28)**	**p[2]**
**Chao1**	24.00 (19.00 – 27.00)	23.50 (19.50 – 28.25)	17.00 (15.00 – 22.00)	0.002[a]
**Shannon**	2.01 (1.62 – 2.18)	1.72 (1.50 – 2.21)	1.65 (1.09 – 1.82)	0.021[b]

1. Values expressed as median and 25th and 75th percentiles.
2. Kruskal-Wallis test, followed by Dunn's test for multiple comparisons:
   a. HMO x GOS/FOS p>0.05; **HMO x BM p<0.05; BM x GOS/FOS p<0.05;**
   b. HMO x GOS/FOS p>0.05; **HMO x BM p<0.05;** BM x GOS/FOS p>0.05**.**

Table S6. Alpha-diversity indices in the fecal microbiota at the final collection in the three studied groups

	GROUP			
**Indice**	**HMO (n= 29)**	**GOS/FOS (n= 30)**	**BM (n= 28)**	**p[2]**
**Chao1**	26.50 (23.25 – 30.75)	26.50 (22.50 – 34.25)	20.50 (16.00 – 24.75)	<0.001[a]
**Shannon**	2.09 (1.70 – 2.31)	2.18 (1.89 – 2.47)	1.65 (1.27 – 1.98)	<0.001[a]

1. Values expressed as median and 25th and 75th percentiles.
2. Kruskal-Wallis test, followed by Dunn's test for multiple comparisons:
   a. HMO x GOS/FOS p>0,05; **HMO x BM p<0,05; BM x GOS/FOS p<0,05.**

Table S7. Percentile indices and Z-scores related to the anthropometric measurements of the investigated groups at the end of the study

Indices	GROUP			p^2
	**HMO (n=29)**	**GOS/FOS (n=30)**	**BM (n=29)**	
**Weight / Length (W/L)**				
Percentile	48.5 ± 16.40	41.5 ± 18.13	41.4 ± 18.80	0.227
Z Score	-0.0 ± 0.45	-0.2 ± 0.64	-0.3 ± 0.70	0.239
**Weight / Age (W/A)**				
Percentile	51.9 ± 14.4	42.5 ± 18.24	41.1 ± 18.25	0.036[a]
Z Score	0.0 ± 0.39	-0.2 ± 0.56	-0.2 ± 0.53	0.041[b]
**Length / Age (L/A)**				
Percentile	55,6 ± 22,19	50,3 ± 23,28	49,1 ± 23,36	0,536
Z Score	0,1 ± 0,68	-0,0 ± 0,78	-0,0 ± 0,68	0,559
**BMI / Age (B/A)**				
Percentile	48,1 ± 13,67	40,0 ± 18,00	39,5 ± 18,44	0,097
Z Score	-0,0 ± 0,37	-0,3 ± 0,57	- 0,3 ± 0,63	0,102
**Head Circumference / Age (HC/A)**				
Percentile	56,6 ± 23,75	57,3 ± 23,64	57,1 ± 26,61	0,995
Z Score	0,2 ± 0,70	0,2 ± 0,72	0,2 ± 0,89	0,994

1. Values expressed as mean ± SD (standard deviation). One-way ANOVA analysis.
2. Chi-square test. Significance level of *p <0.05:
   a. HMO x GOS/FOS p=0.094; **HMO x BM p=0.041;** BM x GOS/FOS p=0.942;
   b. HMO x GOS/FOS p=0.091; HMO x BM p=0.058; BM x GOS/FOS p=0.975.
